# 

**Published:** 1877-06

**Authors:** 


### CONTENTS.

COMMUNICATIONS.

Diseased Gums—*L. C. Ingersol*.................... 229

Principles involved in Filling Teeth with Gold—*S. B. Palmer*.......... 239

Dental Societies—*K. B. Davis*.................... 242

SELECTIONS.

The Homology of the Dental Tissues, etc.,—*A. H. Thompson*.......... 249

Gingivitis in Pregnancy.................... 258

Vitalism and Vital Medication—*R. C. Bond*.................... 259

EDITORIAL.

Local Anaesthesia.................... 265

The Diamond Disk.................... 265

Societies.................... 265

Biographical.................... 266

Pennsylvania State Dental Society.................... 268

Indiana State Dental Association.................... 269

Tennessee Dental Society.................... 270

Southern Dental Association.................... 271

Erratum.................... 271

Conneticut Valley Dental Society.................... 272

